# Regulatory flexibility under pressure: an asymmetric brain-body model of leadership in high-stakes teams

**DOI:** 10.3389/fnins.2026.1957101

**Published:** 2026-09-24

**Authors:** Markus M. Luedi, George Kohlrieser

**Affiliations:** 1 Department of Anaesthesiology, Rescue and Pain Medicine, HOCH Health Ostschweiz, Cantonal Hospital of St. Gallen, St. Gallen, Switzerland; 2 Department of Anaesthesiology and Pain Medicine, Inselspital, Bern University Hospital, University of Bern, Bern, Switzerland; 3 IMD Business School, Lausanne, Switzerland

**Keywords:** allostasis, autonomic regulation, brain–body axis, interoception, leadership, physiological coupling, psychological safety, stress

## Abstract

Leadership under pressure is often framed as a cognitive challenge, although consequential decisions are made by embodied agents whose cardiovascular, respiratory, autonomic, endocrine, and interoceptive states change with stress. We propose an asymmetric interpersonal brain-body model for high-stakes teams. Contextual demands alter a leader’s regulatory state; this state shapes cognitive flexibility, social perception, communication, and action; and leader signals modify the team’s regulatory environment and willingness to disclose uncertainty, dissent, revise plans, or withdraw. These responses feed back to the leader. The model builds on established evidence that bodily signals contribute to subjective feeling and decision-making, stress reorganizes large-scale neural processing, and social partners can alter neural and endocrine responses to threat. Its novel claim is narrower: hierarchy makes interpersonal regulation directionally asymmetric, linking brain-body dynamics to information flow and collective decision quality. Regulatory flexibility, i.e., context-sensitive mobilization and recovery, is proposed as more informative than tonic calm or physiological synchrony. A four-region color heuristic is retained as a translation layer, not as a neurobiological taxonomy. We specify competing explanations, failure criteria, and multimodal within-person and team-level designs for testing the framework in high-stakes teams and crisis simulations.

## 1 Introduction

An old parable asks three practical questions: Who matters most now? What matters most now? What action matters most? Under pressure, leaders may remember the questions yet lose the attentional breadth, cognitive flexibility, and social receptivity required to answer them well (Luedi, 2022). This is visible, for example, in perioperative and acute-care medicine, where distributed information, hierarchy, time pressure, and potentially irreversible consequences coexist. Technical expertise remains indispensable, but stress changes how uncertainty and dissent are perceived and acted upon (Luedi and Urman, 2021).

Brain–body neuroscience provides a mechanistic basis for this observation. Activity in the insular and cingulate systems contributes to representation of visceral responses and interoception (Critchley et al., 2004), while the sensing and regulation of bodily signals spans cardiovascular, respiratory, endocrine, metabolic, and immune channels (Khalsa et al., 2018). Acute stress can reorganize large-scale neural networks toward salience and vigilance (Hermans et al., 2011), and psychosocial stress can reversibly disrupt prefrontal attentional control (Liston et al., 2009). Leadership under pressure is therefore not simply cognition followed by bodily expression; it is cognition occurring within changing bodily conditions. At a conceptual level, the present framework was inspired by Porges’ Polyvagal Theory, particularly its proposition that autonomic state shapes the availability of social engagement, defensive mobilization, and withdrawal (Porges, 2001). In our polyvagal world, Porges emphasizes neuroception, the nervous system’s largely non-conscious evaluation of cues of safety and threat, and the role of social connection in co-regulation (Porges and Porges, 2023). These ideas offer a useful translational bridge to leadership: prosody, facial expression, predictability, and relational presence may function as cues that widen or narrow a team member’s capacity to remain engaged. However, the proposed model does not depend on a one-to-one correspondence between these functional patterns and specific vagal circuits, nor does it adopt the theory’s anatomical or evolutionary claims as established mechanisms.

This Perspective addresses a specific scientific gap. Recent social-neuroscience frameworks already conceptualize interaction as multilevel coupling among neural, autonomic, interoceptive, and behavioral processes (Lettieri, 2026). While we do not relabel that general framework as leadership, we extend it to asymmetric roles in which one person disproportionately controls attention, turn-taking, escalation, and the interpersonal cost of disagreement. Our outcome is not synchrony itself, but whether regulatory dynamics alter information flow, adaptive plan revision, error detection, and collective decisions in high-stakes teams. The claims are tiered: within-person brain–body pathways are established; social transmission and regulation are supported but context dependent; the directional leader–team loop is the novel, falsifiable proposal.

## 2 Scientific premise: regulation before representation

A linear account assumes that a leader evaluates information and then expresses a decision through the body. A recursive account begins with appraisal of goals, controllability, uncertainty, identity, and prior experience. Central autonomic and neuroendocrine responses alter vascular tone, cardiac and respiratory dynamics, metabolic mobilization, and attention. Afferent signals are then integrated with external evidence and expectations, contributing to what the situation feels like and which actions appear available (Critchley and Garfinkel, 2017; McEwen, 1998).

Interoception is not a single sense or a synonym for conscious heartbeat detection. It includes sensing, interpretation, integration, and regulation of internal signals (Khalsa et al., 2018). Bodily activation does not determine behavior: a similar cardiac or visceral change may be interpreted as readiness, urgency, threat, or loss of control. Interoceptive ability can improve or impair intuitive decisions depending on whether bodily predictions are informative for the task, showing why “greater body awareness” is not uniformly beneficial (Dunn et al., 2010).

Stress also changes network configuration and executive availability. Noradrenergic activation during acute stress increases coupling within a distributed salience network (Hermans et al., 2011), whereas sustained psychosocial stress disrupts frontoparietal processing and attentional control (Liston et al., 2009). These findings support a shift in processing priorities, not a simple loss of rationality. Immediate mobilization may be adaptive when action is unambiguous; the same narrowing may be costly when information is distributed and plans must be revised.

The relevant construct is regulatory flexibility: the capacity to deploy, modify, and recover responses according to context (Bonanno and Burton, 2013). To keep the construct falsifiable, we define it through three core dynamic dimensions only: context-sensitive reactivity, adaptive switching, and recovery. Information sampling, receptivity to contradiction, and plan revision are behavioral readouts of switching rather than additional dimensions; motivational orientation is treated as a moderator or descriptive axis, not as part of the flexibility score. High flexibility is therefore not tonic calm but an event-appropriate response, timely behavioral change when evidence changes, and efficient recovery without loss of task performance. Low flexibility is expressed as poorly matched or persistent mobilization or withdrawal, delayed switching despite disconfirming information, and/or slow recovery. These are episode-level, within-person properties relative to predefined perturbations and baselines, not personality labels. Heart-rate variability and other biomarkers may index parts of this process, but they are influenced by respiration, posture, medication, fitness, age, and analysis choices and cannot diagnose leadership quality (Laborde et al., 2017). Table 1 specifies measurable variables, timescales, expected patterns, and candidate analyses.

**Table 1:** Operationalization of the three core dimensions of regulatory flexibility.

Core dimension	Observable variable	Timescale	Expected pattern with higher flexibility	Candidate analysis
Context-sensitive reactivity	Event-related change in cardiac/heart rate variability, electrodermal activity, respiration, pupil and subjective arousal, indexed to a predefined perturbation	Seconds to minutes	Response magnitude matched to task demand; not uniformly low; task performance remains available	Event-aligned multilevel models; within-person response functions
Adaptive switching	Latency/probability of broadening questions, sampling new information, accepting contradiction, reallocating attention or revising a plan after a disconfirming cue	Seconds to minutes	Shorter evidence-contingent switch latency and greater probability of justified revision after new information	Multilevel logistic or survival models; change-point analyses
Recovery	Return of cardiac, respiratory, electrodermal activity and state-report measures toward individual baseline after perturbation; endocrine recovery analysed on its slower timescale	Tens of seconds to minutes; hypothalamic-pituitary-adrenal axis tens of minutes	More efficient return toward baseline without rebound, withdrawal or performance cost	Nonlinear decay/time-to-recovery models; state-space models

Three peripheral channels make the topic’s brain–body relevance concrete. Cardiac and vascular afferents contribute to central representations of bodily arousal, including insular and cingulate activity implicated in subjective feeling (Critchley et al., 2004; Craig, 2002). Respiration is both autonomic output and sensory input; nasal breathing can entrain limbic oscillations and modulate performance in laboratory tasks (Zelano et al., 2016). Endocrine responses, particularly hypothalamic–pituitary–adrenal activation, evolve more slowly and may persist after the precipitating event. These channels interact but should not be collapsed into a unitary “regulated state.” Concordance may occur, but dissociation across cardiac, respiratory, endocrine, behavioral, and subjective measures is theoretically informative.

Timescale is equally important. Cardiac and respiratory changes may shape seconds-long exchanges; hypothalamic–pituitary–adrenal signaling and metabolic mobilization influence longer episodes; sleep loss, inflammation, and cumulative allostatic load constrain recovery across days or months (McEwen, 1998). A brief defensive state may be adaptive during immediate danger, while repeated episodes under chronic load can become self-reinforcing through expectations, habits, and team responses. The model therefore distinguishes momentary state, short-term recovery, and longer-term regulatory capacity. This temporal distinction prevents an acute-state account from becoming an explanation for every leadership failure.

## 3 From individual state to asymmetric social regulation

A leader’s state becomes socially legible through prosody, speech rate, gaze, posture, response latency, interruption, humor, tolerance of silence, and the questions asked. These signals help team members estimate whether uncertainty may be acknowledged and disagreement is safe. Communication therefore carries regulatory information as well as semantic content.

High-profile social-neuroscience findings establish plausibility but not the proposed mechanism. Supportive hand-holding attenuates neural responses to threat, demonstrating that social relationships can change individual threat regulation (Coan et al., 2006). Speaker-listener neural coupling tracks successful communication (Stephens et al., 2010), and interpersonal neural synchronization has been associated with emergent leadership in small-group discussion (Jiang et al., 2015). Real-world classroom synchrony tracks engagement, but is likely influenced by shared attention and common input (Dikker et al., 2017). Stress responses can also be socially transmitted: observers may show cortisol increases when watching another person undergo stress, moderated by closeness and observation conditions (Engert et al., 2014).

These studies do not show that a leader’s physiology causes a team’s behavior. Neural or physiological synchrony can reflect shared stimuli, task structure, movement, mutual prediction, common stress, or genuine co-regulation; synchrony is neither directional nor intrinsically beneficial (Lettieri, 2026; Palumbo et al., 2017). Recent triadic-team research is especially cautionary: predictability of a teammate’s future actions from others’ behavioral and physiological data forecast performance, whereas simple physiological and behavioral synchrony showed limited association with performance (Qin et al., 2025). The scientifically relevant target may therefore be coordinated predictability and adaptive information exchange, not maximal alignment.

The evidence cited here supports different claims with different strength. Hand-holding, empathic stress, speaker-listener coupling, and classroom studies support the plausibility of interpersonal transmission or shared dynamics, but not hierarchical asymmetry. The association between neural synchronization and emergent leadership links interpersonal coupling to leadership emergence, yet does not manipulate formal authority (Jiang et al., 2015). Nembhard and Edmondson show that leader inclusiveness and professional status influence psychological safety and improvement behavior in healthcare teams (Nembhard and Edmondson, 2006), providing behavioral evidence that status can create asymmetric access to the decision process. Among the evidence cited here, however, none demonstrates that the physiology of a formally assigned leader exerts a disproportionately strong regulatory effect on team members. That physiological asymmetry is the novel hypothesis to be tested, not an empirical premise. Accordingly, we use three terms with increasing evidentiary burden. Physiological coupling denotes cross-person covariation or statistical dependence and carries no causal interpretation. Interpersonal influence requires that a change in one person’s state or behavior prospectively predicts change in another after accounting for each person’s prior state, shared task events, movement, speech, and other common inputs. We reserve co-regulation for interaction in which such influence demonstrably changes the other person’s regulatory trajectory through an identifiable social pathway. For the present model, a strong claim of leader-to-team co-regulation would require temporal precedence, shared-stimulus or yoked controls, evidence that opening or blocking communication changes the effect, and preferably manipulation of formal authority or role; behavioral mediation should link leader state to team response. Lead–lag association alone is insufficient.

Our extension is asymmetric and recursive. Pressure changes the leader’s regulatory state; the state shapes communication and action; team members respond through disclosure, dissent, compliance, reassurance, or withdrawal; and those responses alter the leader’s subsequent appraisal. Silence may be mistaken for agreement and intensify control, whereas early disclosure may distribute uncertainty and support recovery. Power moderates the loop because leaders usually control turn-taking, escalation, evaluation, and the consequences of speaking. Hierarchy thus changes not only who influences whom, but which signals can safely enter the collective decision process. Figure 1 summarizes this asymmetric brain–body loop, distinguishing established within-person pathways from the proposed leader-team feedback process and its principal moderators.

**Figure 1:**
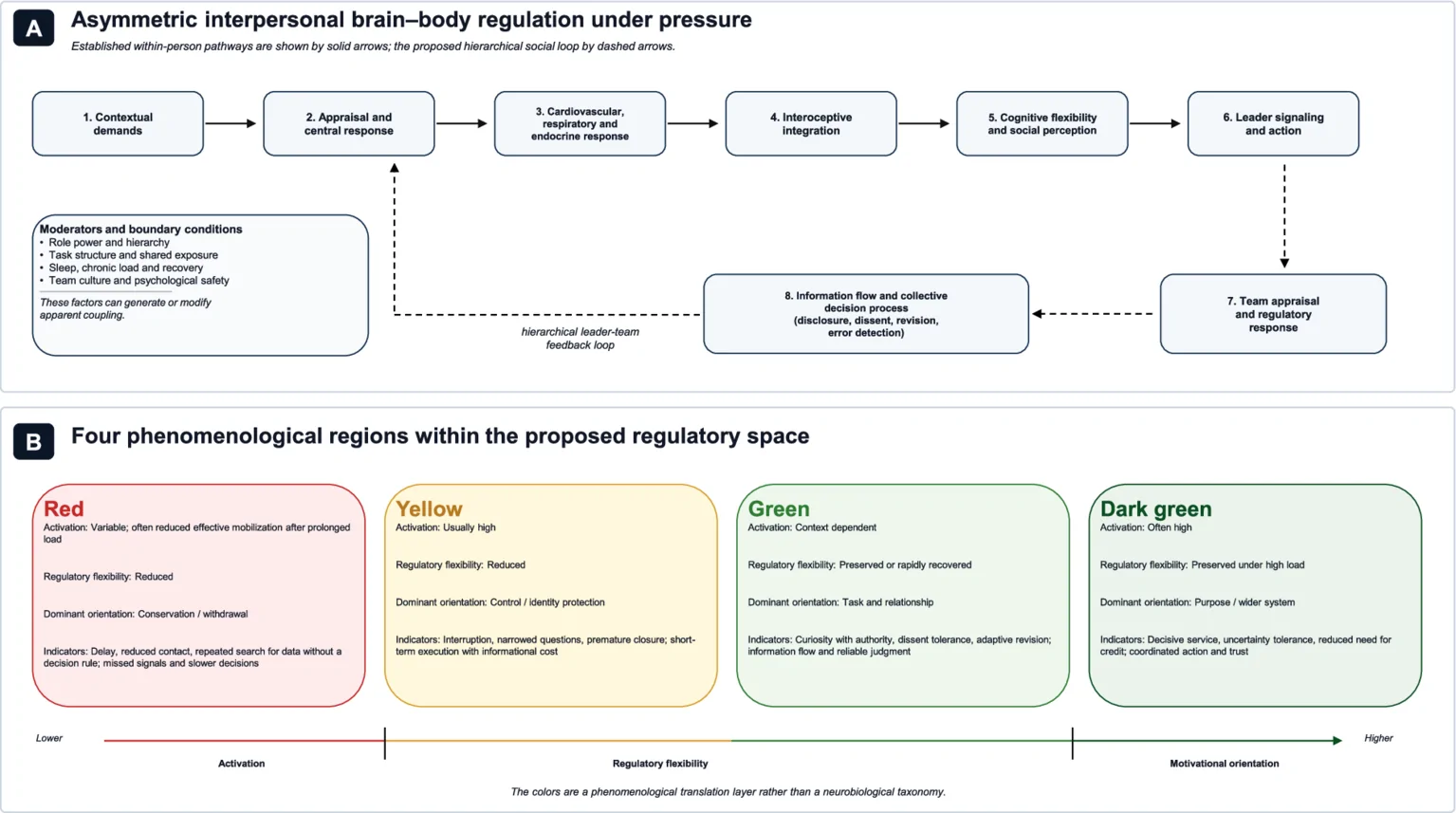
Asymmetric interpersonal brain–body regulation under pressure. Contextual demands influence appraisal and central regulatory responses, which shape cardiovascular, respiratory, and endocrine activity, interoceptive integration, cognitive flexibility, social perception, and leader signaling and action. Solid arrows indicate established or well-supported within-person pathways. Dashed arrows represent the proposed interpersonal hypothesis and are mediated by observable leader signaling and communication rather than implying a direct physiology-to-physiology pathway: leader regulatory state influences signaling behavior; signaling behavior alters team appraisal, disclosure, dissent, plan revision, error detection, and collective decision-making; and team responses subsequently feed back to the leader. Physiological coupling may accompany interaction but does not by itself constitute interpersonal influence or co-regulation. Formal authority and role power, actual speaking time and information content, expertise and perceived status, task structure, shared exposure, sleep, chronic load, recovery, team culture, and psychological safety are moderators, alternative explanations, or boundary conditions. The figure does not encode the four color regions as biological states; those remain only as a secondary communication heuristic in Table 3.

**Table 3:** Secondary phenomenological communication heuristic mapped onto continuous regulatory dimensions.

Region	Activation	Regulatory flexibility	Dominant orientation	Observable indicators and predicted consequence
Red	Variable; often reduced effective mobilization after prolonged load	Reduced	Conservation/withdrawal	Delay, reduced contact, repeated search for data without a decision rule; missed signals and slower decisions
Yellow	Usually high	Reduced	Control/identity protection	Interruption, narrowed questions, premature closure; short-term execution with informational cost
Green	Context dependent	Preserved or rapidly recovered	Task and relationship	Curiosity with authority, dissent tolerance, adaptive revision; information flow and reliable judgment
Dark green	Often high	Preserved under high load	Purpose/wider system	Decisive service, uncertainty tolerance, reduced need for credit; coordinated action and trust

The same terse instruction may regulate a team during immediate resuscitation and silence it during diagnostic uncertainty. The model therefore predicts strongest leader effects when hierarchy is salient, knowledge is distributed, and surfacing disconfirming evidence is consequential. It predicts weaker effects when tasks are fully scripted, information is centralized, or team members can challenge decisions without interpersonal cost. Repeated loops may consolidate into culture and psychological safety (Edmondson, 1999; Nembhard and Edmondson, 2006). In Secure Base Leadership terms, leaders provide both protection and permission to explore: a relational base from which team members can disclose uncertainty, challenge assumptions, and take appropriate interpersonal risks (Kohlrieser et al., 2012). A parallel is visible in hostage negotiation, where influence depends on preserving contact and dialog rather than escalating control (Kohlrieser and Kohlrieser, 2024). These leadership frameworks are offered as conceptual parallels to asymmetric access and dialog, not as evidence for a physiological mechanism. This focus on asymmetric access to the decision process is the principal distinction from general accounts of interpersonal brain–body coupling.

## 4 Secondary translation layer: four phenomenological regions

The color framework originated as a reflective account of inner states (Luedi, 2026). Although conceptually influenced by polyvagal distinctions among social engagement, defensive mobilization, and withdrawal (Porges, 2001), the regions are phenomenological heuristics rather than physiological equivalents of proposed polyvagal states. Here they are retained only as a secondary communication layer over continuous dimensions. The scientific model, predictions, operationalization (Table 1), and competing models (Table 2) are stated entirely without color labels. Activation and the three core flexibility dimensions are considered first; motivational orientation is an adjacent descriptive axis. Similar behavior may arise through different physiological pathways, and no region maps uniquely onto a neural circuit, autonomic branch, hormone, or developmental stage.

**Table 2:** Competing models and empirical patterns that discriminate among them.

Competing model	Key prediction	Discriminating empirical pattern	Interpretation
Context-only	Shared task events and exposure generate apparent cross-person coupling	Leader–team covariance largely disappears after event, movement, speech and shared-stimulus controls	No interpersonal regulatory mechanism required
Behavior-mediated	Leader state matters only because it changes observable communication	Sequential mediation is supported and residual physiology-to-team effects add no predictive value	Brain–body dynamics may matter within the leader; direct interpersonal physiological coupling is unnecessary
Symmetric interpersonal coupling	Influence is reciprocal and not specifically hierarchical	Leader → member and member → leader effects are comparable after role/context controls and do not reverse with authority reassignment	General coupling model preferred over asymmetric leadership model
Asymmetric brain–body	Formal authority amplifies leader → team influence beyond context and communication alone	Leader → member effects remain incrementally predictive, strengthen when hierarchy/interpersonal cost is salient, and attenuate or reverse with role reversal	Supports the proposed extension

Red denotes withdrawal when demands exceed available capacity; yellow denotes defensive mobilization organized around control or identity protection; green denotes context-sensitive engagement with preserved or rapidly recovered flexibility; and dark green denotes preserved flexibility under high load combined with stronger orientation toward patient, team, or system purpose (Baima et al., 2026; Luedi, 2025a). Red and yellow may be adaptive. Concern arises when they persist beyond their usefulness, remain unrecognized, or repeatedly shift costs to the team. Chronic workload and burnout can narrow the capacity for recovery (Milenovic et al., 2020). Observable composure is not equivalent to regulation: distress may be expressed while flexibility is preserved, or concealed while cognition remains rigid. The testable question is not “Which color is the leader?” but how the three flexibility dimensions change across episodes and what happens to information flow. Dark green is not a higher neurobiological state; it is a normative translational label for preserved flexibility plus system-oriented goal framing and must not be used as a scientific endpoint. Table 3 therefore presents the colors only as an optional communication heuristic.

## 5 A falsifiable research program

The model must outperform simpler explanations such as personality, workload, role power, task difficulty, and observable communication alone. Four predictions provide a starting point.

Prediction 1: within-person regulatory dynamics add explanatory value. Repeated measures should show that changes within the same leader predict interruption, receptivity to contradiction, information search, and plan revision after adjustment for sleep, workload, expertise, task difficulty, and stable traits. If physiology and state reports add no value beyond behavior and context, the brain–body component should be reduced rather than retained rhetorically.

Prediction 2: recovery dynamics matter more than tonic calm. Effective episodes should not correlate monotonically with lower heart rate, cortisol, electrodermal activity, or subjective stress. Better outcomes should be associated with context-sensitive mobilization, preserved behavioral switching, and faster recovery after disruption. Biomarker differences without mediation through decision or communication processes would not support the proposed mechanism.

Prediction 3: leader–team effects are directional and context dependent. Multilevel time-series models should test whether leader changes precede team silence or speaking up, whether team uncertainty precedes controlling behavior, and when reciprocal effects occur. Simultaneous measures could combine ECG, respiration, electrodermal activity, pupillometry, voice acoustics, movement, behavioral coding, and brief state reports. Integration should be staged rather than forcing convergence: first, modality-specific event-aligned trajectories should be modeled at their native timescales; second, a dynamic latent regulatory state should be tested only if the covariance structure supports it and if it improves out-of-sample prediction over modality-specific and behavior-only models; third, cross-lagged or state-space analyses can examine directional relations across persons. Discordance is informative—for example, rapid autonomic recovery with sustained endocrine activation—and should not be treated as measurement failure. Recent operating-room studies demonstrate the feasibility of combining neurophysiological, autonomic, endocrine, subjective, behavioral, and inter-person measures in real procedures while also highlighting role specificity, non-stationarity, and unequal temporal resolution across modalities (Ronca et al., 2026; Ronca et al., 2025). Shared exposure, speech content, motor activity, and task events must be modeled explicitly; otherwise apparent coupling remains ambiguous.

Prediction 4: perturbations improve collective performance only when they improve flexibility. State labeling, paced breathing, attentional redirection, purpose-based reappraisal, explicit invitations to dissent, or structured pauses should be compared with active controls. Outcomes should include disclosure, adaptive revision, error detection, and recovery, not relaxation alone. Failure to change these mediators despite greater subjective calm would weaken the model.

Operationalization should be event-based and preregistered. Perturbations such as a new contradiction, a sudden change in patient state, time pressure, or an explicit challenge should define event onset; individual baseline windows should be specified *a priori*. The three core dimensions in Table 1 should be analysed separately in primary analyses. Any composite or latent flexibility score is secondary and should be used only when a preregistered measurement model demonstrates adequate structure, temporal stability, and out-of-sample validity. Post-hoc classification of an episode as “flexible” because the outcome was good should be prohibited. Blinded behavioral coding, temporal validation, and out-of-sample prediction are needed to avoid circularity. Laboratory simulations can manipulate ambiguity, social evaluation, time pressure, and role power; field studies can sample handovers, crises, and unexpected changes in plan. Operating rooms, emergency departments, intensive care units, rescue services, and crisis simulations are strong settings because physiological stressors, interdependent roles, and meaningful outcomes converge.

Causal inference requires more than lagged correlation. A decisive factorial design would cross formal authority (assigned leader versus team member) with communication behavior (experimentally standardized higher versus lower speaking time or informational control), hold expertise and task information as constant as feasible, and reverse roles across repeated rounds. If hierarchy itself contributes explanatory value, leader-to-member effects should persist after actual speaking time, information content, perceived status, expertise, personality, and communication style are modeled, and the directional effect should attenuate or reverse when authority assignment is reversed. If effects instead follow speaking time or task-relevant information regardless of formal role, the hierarchy claim is weakened. Yoked teams exposed to the same events without direct interaction can estimate covariance attributable to shared stimuli.

The primary mediation hypothesis is sequential: leader regulatory state at time t → observable signaling behavior at t + 1 → team disclosure or dissent at t + 2 → plan revision, error detection, or collective decision quality. Candidate behavioral bridges include fundamental frequency and prosody, speech rate and amount, interruption rate, response latency, question openness, turn-taking, and linguistic markers of uncertainty. In air-traffic control, speech amount and fundamental frequency changed with stress alongside neurophysiological measures, demonstrating the feasibility of integrating voice and physiology without establishing a leadership mechanism (Borghini et al., 2020). Sequential multilevel mediation or dynamic structural-equation models should test whether communication carries the effect. A residual direct physiology-to-team pathway is not required by the framework and should not be retained if observable behavior fully mediates the association.

Competing explanations should be preregistered rather than treated only as covariates (Table 2). A context-only model predicts that cross-person covariance disappears after modeling task events and shared exposure. A behavior-mediated model predicts that leader physiology or state affects the team only through observable communication, with no residual cross-person physiological pathway. A symmetric coupling model predicts comparable member-to-leader and leader-to-member effects after role and context controls. The proposed asymmetric brain–body model predicts incremental leader-to-member effects beyond context and behavior, amplification when formal authority and the interpersonal cost of disagreement are high, and attenuation or reversal with authority reassignment or role reversal. If simpler models perform as well or better out of sample, they should be preferred.

No single analysis establishes causality. Convergence across experimental manipulation, temporal ordering, behavioral mediation, shared-stimulus controls, role reversal, and replication is required before describing the process as asymmetric co-regulation.

Ethical limits are integral, not ancillary. Physiological data must not be used to label leaders as biologically fit, infer moral character, or enable punitive surveillance. Individual baselines, medication, health, age, sex, culture, and context require consideration. The acceptable scientific aim is to understand dynamic mechanisms and improve systems, not to rank people by a supposed regulatory phenotype.

## 6 Translational implications

Three low-burden practices can serve as experimental perturbations, not established treatments. Somatic Experiencing, Jungian individuation, and Internal Family Systems are retained only as brief conceptual parallels in this translational section and are not components of the scientific model or sources of mechanistic predictions. Somatic Experiencing uses graded attention to interoceptive, proprioceptive, and kinesthetic signals (Payne et al., 2015); Jungian and Internal Family Systems traditions offer reflective language for noticing defensive, controlling, or withdrawing reactions without equating them with the whole person (Jung, 2014; Schwartz, 2013; Schwartz and Sweezy, 2019). In this Perspective, these parallels motivate only non-clinical practices for metacognitive distance and behavioral choice; ordinary occupational pressure is not equated with psychological trauma.

First, naming an emerging pattern, “I am becoming defensive” or “I am withdrawing”, may create metacognitive distance without suppressing emotion. Second, regulating before communicating through a brief pause, slower breathing, or attentional shift may alter both bodily dynamics and message delivery. Nasal respiration can entrain limbic oscillations and modulate cognition (Zelano et al., 2016), while slow-breathing studies show heterogeneous autonomic and psychological effects across protocols and individuals (Zaccaro et al., 2018); neither finding proves improved leadership. Third, reorienting from identity protection toward patient, team, and system purpose may change the motivational context in which activation is interpreted (Luedi, 2025a; Luedi, 2025b).

Individual practices cannot compensate for unsafe staffing, chronic overload, contradictory authority, or punitive culture. Sleep, autonomy, team stability, role clarity, and institutional justice shape regulatory capacity. Brief pre-briefings, explicit invitations to dissent, role clarification, and structured pauses after unexpected information should therefore be evaluated as changes to the social environment, not merely leader skills (Bello et al., 2022). Humor may also be informative: inclusive humor can preserve connection, whereas cynicism may signal defensive distance or depletion, but it remains a contextual behavioral indicator rather than a biomarker (Martens et al., 2026).

## 7 Discussion

This Perspective positions leadership under pressure as an asymmetric, socially embedded brain–body process. Its fit with the brain–body axis lies in explicit pathways linking central appraisal with cardiac, respiratory, autonomic, and endocrine responses, and in the return of peripheral signals to cognition and behavior. The leadership extension asks how those within-person dynamics become social signals that alter another person’s regulatory environment and, through hierarchy, the quality of collective information processing.

The model neither claims physiological determinism nor assigns four colors to neural states. It differs from general multilevel social-coupling accounts by specifying role asymmetry, high-stakes uncertainty, directional feedback, and decision-process outcomes. It also resists the assumption that synchrony equals success. Regulatory flexibility and appropriately timed predictability may be more important than maximal alignment.

The framework would be weakened if regulatory dynamics failed to predict behavior beyond traits, task, and communication; if leader effects disappeared after controlling shared exposure; or if perturbations changed subjective calm without improving disclosure, revision, or error detection. A context-only model would be favored if task events explained nearly all cross-person covariance. A behavior-mediated model would be favored if communication fully carried the association and no residual physiological cross-person pathway improved prediction. A symmetric model would be favored if leader-to-member and member-to-leader effects were comparable after role controls, or if directional effects followed speaking time rather than assigned authority. The asymmetric extension is supported only if role-sensitive models add reproducible out-of-sample explanatory value and respond to authority manipulation or role reversal. These failure conditions are essential because they convert an intuitively attractive narrative into a scientific program. Under pressure, wisdom depends not only on knowing the right questions, but on remaining physiologically and relationally available to ask them, and to serve as a secure base from which others can answer honestly.

## Data Availability

The raw data supporting the conclusions of this article will be made available by the authors, without undue reservation.
